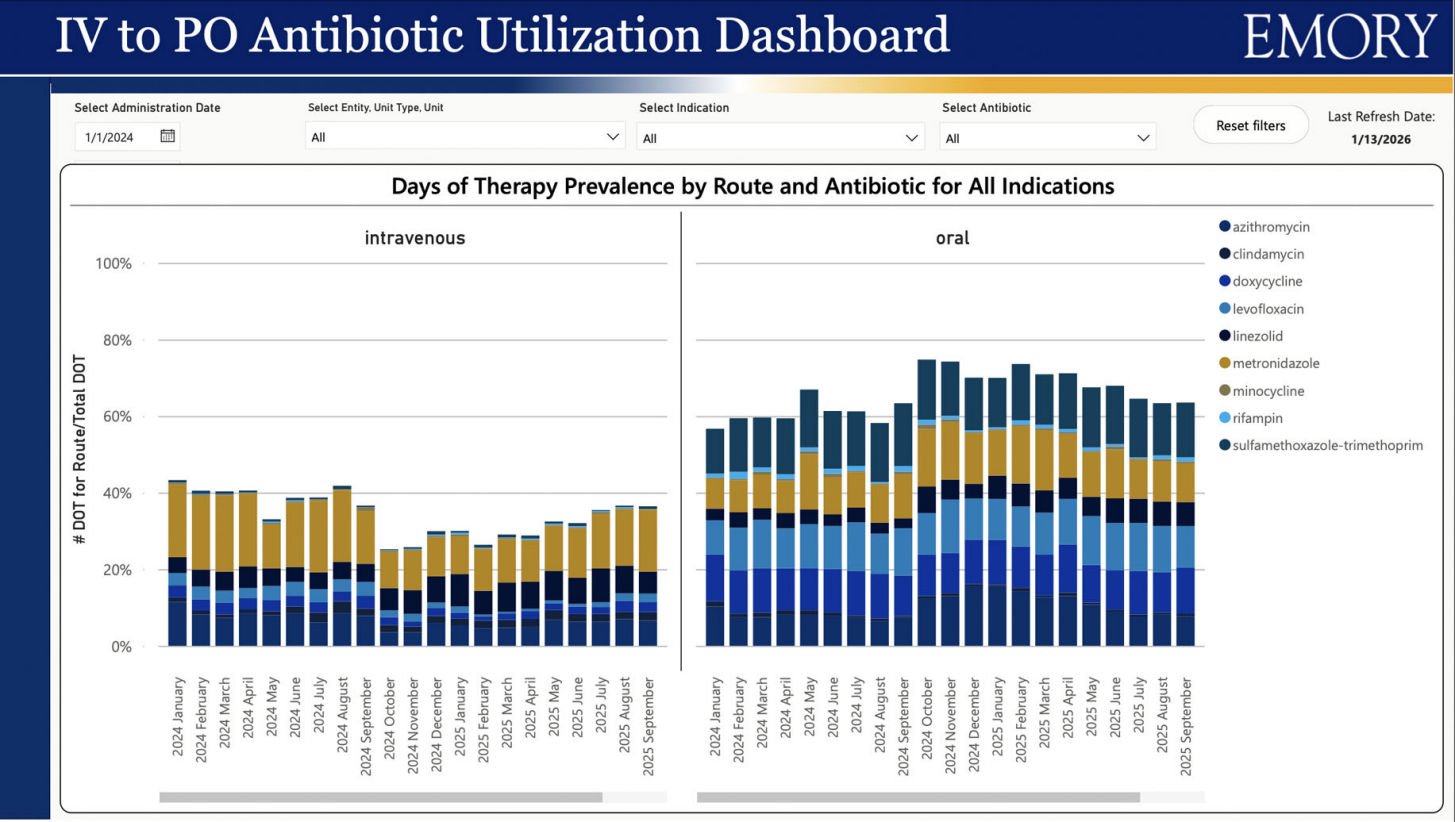# 366 Hard-Wired Habits: The Challenge of De-Prescribing Antibiotics in Critically Ill Patients with CAP

**DOI:** 10.1017/ash.2026.10702

**Published:** 2026-06-23

**Authors:** Jada Guilford, Sujit Suchindran, Kristen Paciullo, Leila Hojat, Sarah Green

**Affiliations:** 1 Emory Healthcare; 2 Emory University School of Medicine; 3 Emory Saint Joseph’s Hospital; 4 Emory University Hospital Midtown; 5 Emory University Hospital

## Abstract

**Background:** Timely conversion of intravenous (IV) antibiotics to oral (PO) therapy remains a core principle of antimicrobial stewardship. Appropriate transition from IV to PO medications reduces the potential for adverse events such as line infections and thrombophlebitis but may also reduce healthcare costs by potentially facilitating a timelier discharge. In addition, IV to PO supports sustainability initiatives by reducing the environmental impact of single-use IV bags and tubing, and PO antibiotics are prepared more quickly by pharmacy and administered easier by nursing, lessening staffing requirements. **Methods:** Emory Healthcare developed a fully automated dashboard to monitor IV to PO transitions and assess adherence to institutional protocols. The dashboard details antibiotic utilization and documented indication for select IV and PO agents among inpatients at Emory Healthcare from January 2024 to the current year-to-date. Selected antibiotics were chosen based on their inclusion in the Emory Healthcare IV to PO Medication Conversion by Pharmacist Protocol. The dashboard integrates data from the electronic health record including antibiotic selection, infection type, and days of therapy. The data is organized into sections and graphs for streamlined viewing and clear interpretation. **Results:** A dashboard was successfully created to monitor IV to PO therapy conversions. The first section (Figure A) shows days of therapy (DOT), which can be filtered by operating unit and indication. The second section (Figure B) displays DOT filtered by route and indication for the specific antibiotics included in the Emory Healthcare IV to PO conversion protocol. The third section (Figure C) displays DOT filtered by route and antibiotic for all indications. The antibiotic and indication charts can additionally be filtered to include any combination of antibiotics, indications, facility, unit type, and units. The dashboard also includes tables with antibiotic route of administration data stratified by facility, unit type (ie. oncology, emergency, operating room, acute care), and individual unit. **Conclusion:** The dashboard provides a novel tool for ASPs to continuously assess utilization and adherence to the Emory Healthcare IV to PO conversion protocol. The dashboard is interactive, customizable, and data is readily available, making it a key tool for optimizing stewardship practices. The data can also be used to identify stewardship opportunities, set quality improvement targets, and quickly display information to key stakeholders.

**Figure:**
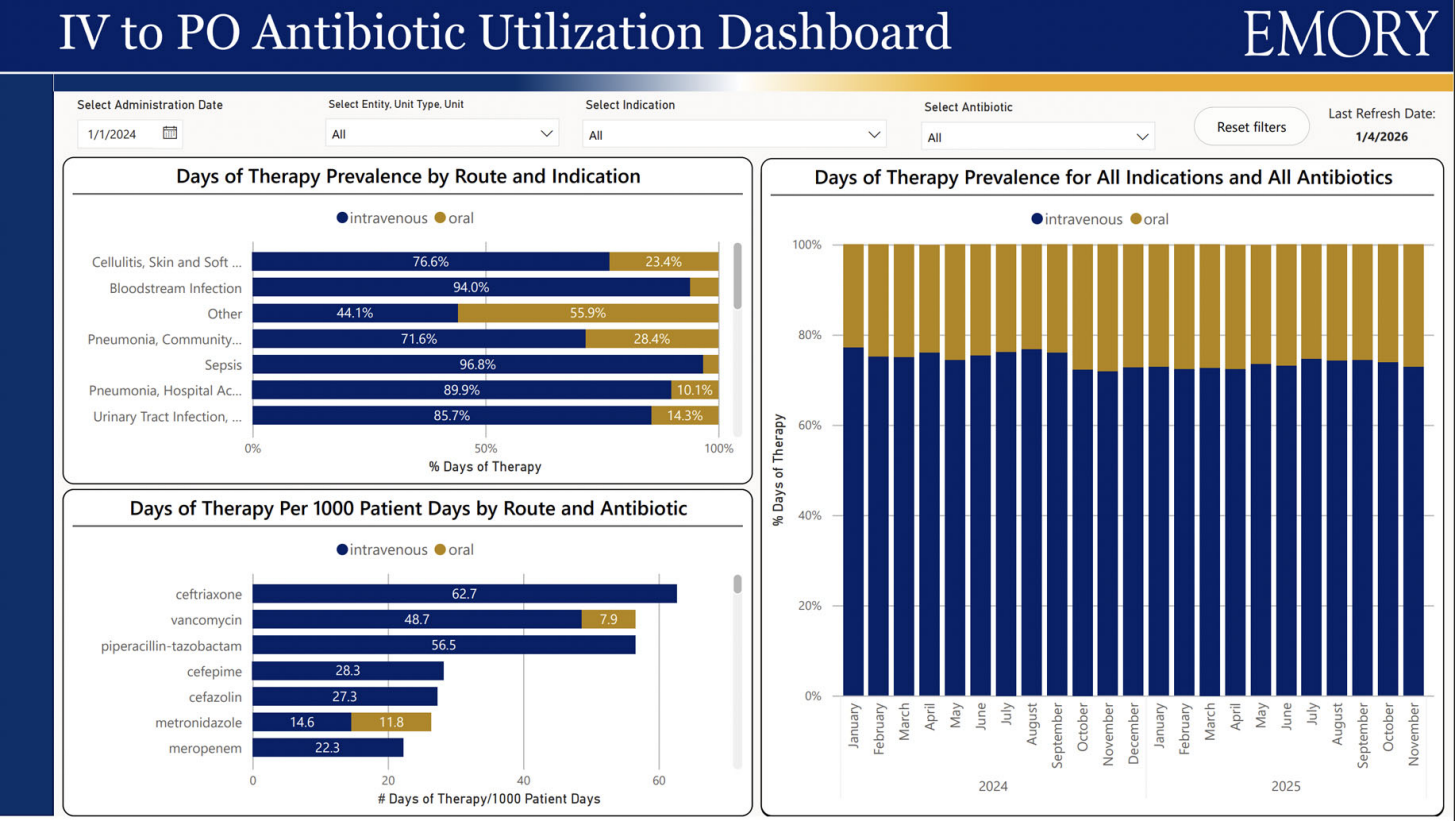

**Figure:**
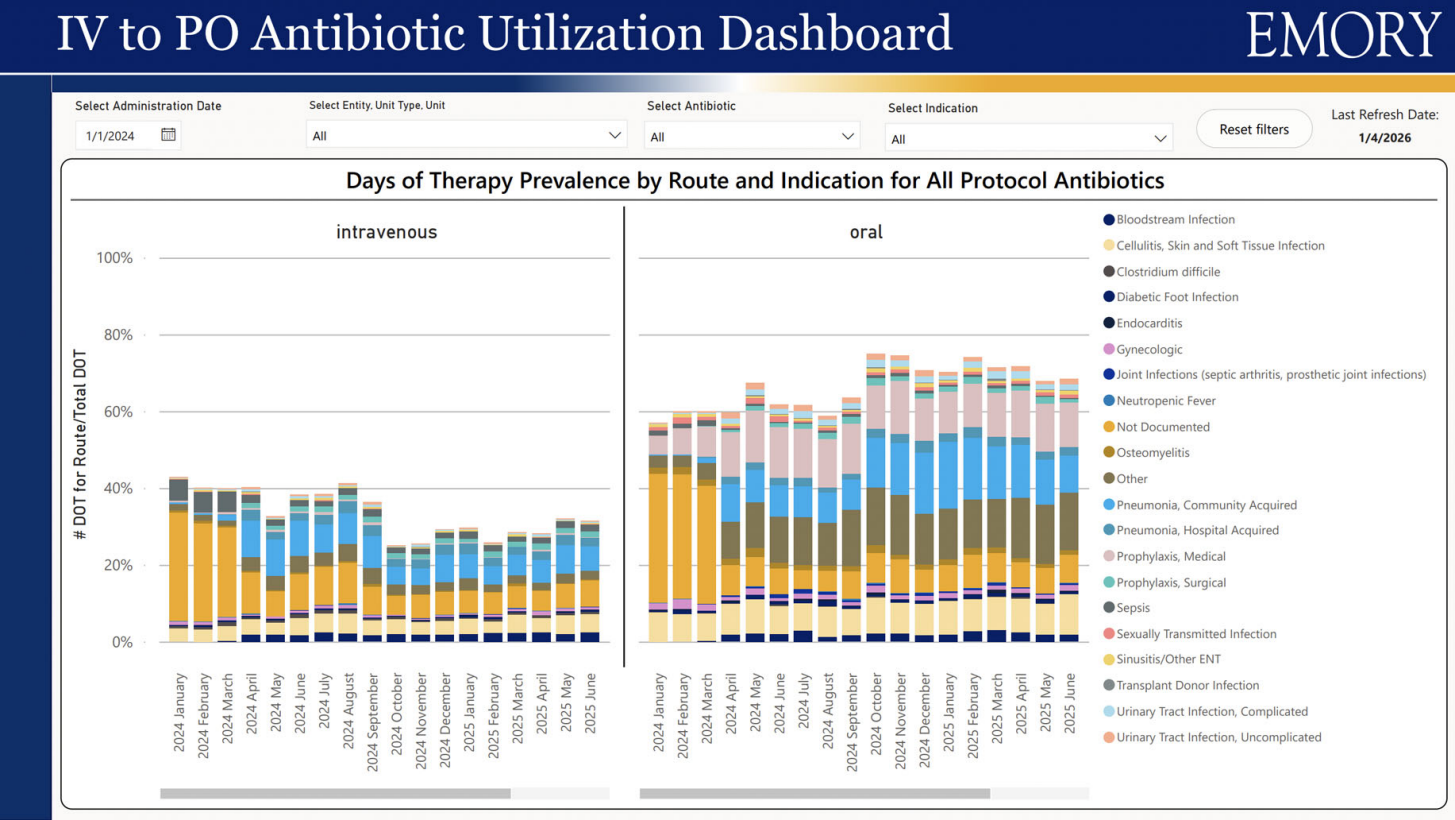

**Figure:**